# Targeting Aberrant Histone Posttranscription Modification Machinery in Esophageal Squamous Cell Carcinoma: Current Findings and Challenges

**DOI:** 10.34133/2022/9814607

**Published:** 2022-08-17

**Authors:** Gang Ma, Tongyang Gong, Zhihua Liu

**Affiliations:** ^1^Department of Gastric Surgery, Tianjin Medical University Cancer Institute and Hospital, National Clinical Research Center for Cancer, Key Laboratory of Cancer Prevention and Therapy, Tianjin, Tianjin's Clinical Research Center for Cancer, Tianjin 300060, China; ^2^State Key Lab of Molecular Oncology, National Cancer Center/National Clinical Research Center for Cancer/Cancer Hospital, Chinese Academy of Medical Sciences and Peking Union Medical College, Beijing 100021, China

## Abstract

Esophageal squamous cell carcinoma (ESCC) is an aggressive malignancy, but the survival rates of patients with ESCC have not improved as yet largely because the available targeted therapies are limited. Histone posttranscription modification (PTM) is a critical epigenetic regulation. Several deregulations in histone PTM machinery have been identified to promote malignant phenotypes of ESCC, providing druggable targets in treating ESCC. Hereby, we briefly describe current progress and challenges ahead in this field.

Esophageal cancer, a commonly diagnostic gastrointestinal malignancy, ranks the sixth for incidence and the fifth for cancer-caused mortality in China [1]. Genomic studies in esophageal squamous cell carcinoma (ESCC), the predominant histological subtype in China, have identified mutations in several genes implicated in epigenetic regulations, such as KMT2D, KMT2C, KDM6A, EP300, and CREBBP, indicating that aberrations in epigenetic machinery play vital roles in ESCC initiation and progression [2].

The N-terminal tails of histone H2A, H2B, H3, and H4 are highly conserved and subjected to diverse posttranscription modifications such as methylation and acetylation of lysine residues. These PTMs modulate the interaction between histone octamers and DNA or bind effector proteins to control chromosome functions, mainly gene expression [3]. A wealth of data has documented that tumor cells exploit this epigenetic machinery to promote the malignant phenotypes *via* yielding aberrant PTMs of histones across human genome and further leading to abnormal activation of oncogenes or repression of tumor-suppressor genes (TSGs) [4]. Deregulated H3K27me3 expression is commonly detected across a wide range of human cancers, including ESCC. A retrospective immunohistochemistry analysis showed that increased global H3K27me3 expression positively correlated with differentiation and lymph node metastasis, as well as poor prognosis in patients with ESCC [5]. Our latest study described the genomic distribution of H3K27me3 in immortalized esophageal epithelial cell line NE2 and ESCC cell line KYSE450, finding that the locations of grand H3K27me3 silencer domains (GSDs) are highly conserved in NE2 cells whereas these GSDs are lost, thereby activating a series of oncogenes (e.g., *TBX20*), in KYSE450 cells [6]. In addition to H3K27me3, the acetylation status of H3K27, which usually marks activated gene expression, is also linked to ESCC. H3K27ac upregulates the expression of lncRNA CCAT1, which further promotes proliferation and motility in ESCC cells [7]. Besides, extensive H3K27ac modification is considered the marker of super-enhancers (SEs), hundreds of which change during ESCC carcinogenesis and metastasis. The gained SEs lead to the increase of *HSP90AA1* and *PDE4B* in ESCC [8]. Additionally, SEs coordinate with transcription factors (TFs), such as TP63 and SOX2, to activate protumor genes expression. TP63 binds SE to increase lncRNA 01503 expression, thereby activating ERK and Akt signaling pathway to boost the aggressive phenotypes in ESCC cells [9]. These results unveil the critical and intricate roles of H3K27 PTMs in regulating gene expressions in esophageal tumorigenesis.

Dozens of enzymes have been identified to add modifications to (“writers”) or remove (“erasers”) them from histones. One primary contribution to aberrations in the histone PTMs is dysfunctional “writers” or “erasers.” The histone demethylase KDM4C, for example, increases in ESCC cell population with enhanced tumor-initiating capability [10]. KDM4C, which is responsible for removing methyl groups from H3K9me2/3, sustains the low level of H3K9 methylation at the promoters of several pluripotency-related genes, such as *SOX2* [10]. More abnormal “writers”/“erasers” and their functions in ESCC are reviewed recently [11]. Whereas these studies shed light on how these enzymes alter histone PTMs to regulate gene expression, there are still some limitations in this research field. Epigenetic regulation is highly dynamic, which means that it changes constantly as tumors evolve. However, most investigations, especially those based on *in vitro* experiments, demonstrate the influence wielded by a given “writer” or “eraser” on the gene expression profile under a specific cellular condition; researchers thereby cannot determine what happened before or will occur afterwards. These described axes of “aberrant writer/eraser-histone PTM-altered expressed gene” to explain aggressive phenotypes in tumors are probably replaced by others under different conditions in tumors. Additionally, the substrates of most “writers” or “erasers” are not limited to histones; thus, researchers hardly determine which substrate(s)-mediated molecular underpinnings are more critical in dictating tumor initiation and progression. One primary cause of these challenges is the lack of proper research techniques at the moment. For example, researchers hardly monitor real-time alterations in global histone PTMs and gene expression profiles simultaneously *in vivo*.

Despite these limitations, the mounting data in this field has prompted the development of small inhibitors of critical molecules in histone PTM machinery and relevant translational research (Table 1). Notably, single targeting of one “writer” or “eraser” usually displays weak tumoricidal effect. In addition to the causes discussed before, another primary reason is probably that inactivation of a single enzyme is compensated by others to catalyze the formation of the same histone PTMs. Therefore, inhibiting more than one target simultaneously is a rational strategy. Junxia Min and colleagues found that inactivation of LSD1 with SP2509 and G9a using UNC0642 at the same time markedly suppressed the propagation in ESCC cells through functional screening an epigenetic library in a panel of cancer cell lines [12]. The authors demonstrated the potent synergistic effect of co-inhibiting LSD1 and G9a in ESCC cells as well as their tumor xenografts [12]. Additionally, the authors discovered that LSD1 and G9a are both deregulated in ESCC, and patients with high expression levels of both LSD1 and G9a had even significantly poor prognosis. All these data indicate that LSD1 and G9a are the potential targets for treating ESCC [12]. Interestingly, the authors showed that dual administration of the two drugs decreased the expression of several genes, such as *PERK*, *ATF4*, *CHOP*, *eIF2α*, and *ATF6*, implicated in ER stress, and targeting ER stress enhanced the sensitivity of ESCC cells to SP2509 and G9a treatment, which provided strong supporting evidence that targeting LSD1, G9a, and ER stress could serve as a promising strategy for clinical treatment of ESCC patients [12]. ER stress is commonly related to cancer therapy. A recent study demonstrated that a newly synthesized compound interacted with alkaline phosphatase (ALP) and reductase, formed nanofibers in the mitochondria of the lung cancer A549 cells to trigger ER stress, and attenuated the cellular proliferation *in vitro* and *in vivo* [13]. Moreover, targeting the key SEs-associated molecules as a therapeutic option in ESCC has been already under investigation. As described before, deregulated SEs control the expression of dozens of genes in favor of ESCC progression. PAK4 expression was increased by SEs in ESCC cells, and pharmaceutical inhibition of this kinase with KPT-9274 remarkably blocked the growth of ESCC cells [14]. HSP90AA1 and PDE4B are two promising druggable targets as single or combined administration of the inhibitor of HSP90AA1 or PDE4B that markedly reduced the proliferation and increased the apoptosis in ESCC cells [8]. Deletion of BRD4 and inhibition of HDAC disrupted the SEs-regulated core regulatory circuitry consisting of TP63, SOX2, and KLF5, thus exerting synergistic antineoplastic effect [15]. These preclinical studies offer promising treatment strategies in ESCC.

In conclusion, the functions of aberrations in histone PTMs machinery remain largely unclear in ESCC, although data in this field is continuously growing. More important is performing high-quality experimentally therapeutic studies to find the rational recipes including epigenetics-based targeted drugs for patients with ESCC.

## Figures and Tables

**Table 1:** Summary of experimentally therapeutic studies based on epigenetics-based drugs in ESCC.

Target	Target function	Drug	Administration strategy	References
LSD1	Lysine demethylase	SP2509	Combined	[12]
G9a	Lysine methyltransferase	UNC0642	Combined	[12]
CDK7	SEs-associated	THZ1	Single	[14]
PAK4	SEs-associated	KPT-9274	Single	[14]
HSP90AA1	SEs-associated	AH	Single/combined	[8]
PDE4B	SEs-associated	ML-030	Single/combined	[8]
BRD4	SEs-associated	ARV-771	Single/combined	[15]
HDAC	SEs-associated	Romidepsin	Single/combined	[15]
